# Supplementary material for: Introducing principles of synaptic integration in the optimization of deep neural networks
Source: Nat Commun. 2022 Apr 7;13:1885. doi: 10.1038/s41467-022-29491-2 (PMC8989917; doi:10.1038/s41467-022-29491-2)
Supplement: Supplementary file 1 — Supplementary Information [file 41467_2022_29491_MOESM1_ESM.pdf]

Supplementary Material
Introducing Principles of Synaptic Integration in the Optimization of Deep Neural Networks

Giorgia Dellaferrera,[1,2] Stanisław Woźniak,[1] Giacomo Indiveri,[2] Angeliki Pantazi,[1] and Evangelos Eleftheriou[1]

[1] *IBM Research – Zurich, Rüschlikon, Switzerland*
[2] *Institute of Neuroinformatics, University of Zurich and ETH Zurich*

Supplementary Figure 1: Applying GRAPES to convolutional layers

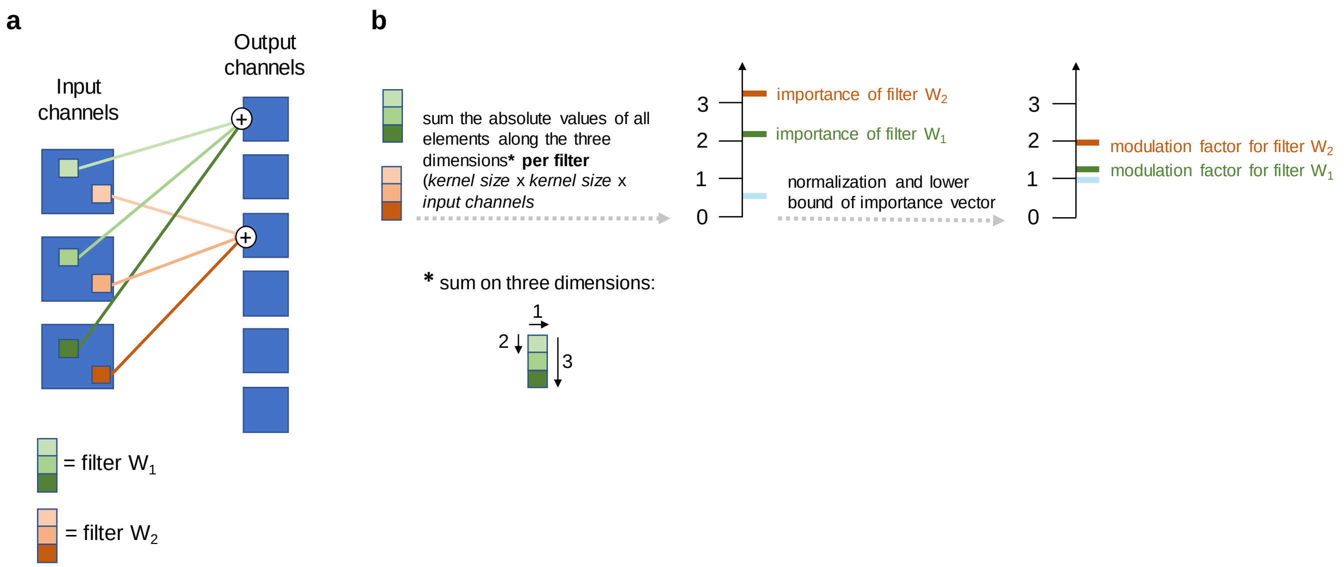

Supplementary Figure 1. Graphical representation of the GRAPES algorithm for convolutional layers. **(a)** Filters of convolutional layers can be grouped according to the output channels, in the same way as weights in fully-connected layers can be grouped based on the post-synaptic node. **(b)** Steps to compute the importance vector and the modulation factors for the convolutional filters starting from the filter elements.

Supplementary Figure 2: The plateau equation for learning curves

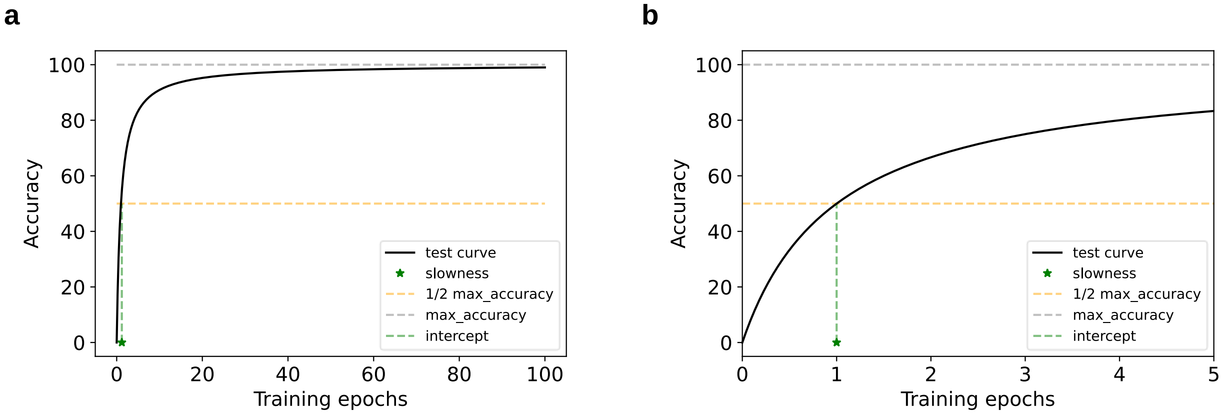

Supplementary Figure 2. Graphical representation of the *Plateau equation for learning curves*: accuracy $= \frac{\text{max\_accuracy} \cdot \text{epochs}}{\text{slowness} + \text{epochs}}$ **(a)** Plateau curve for 100 epochs. From the plot one can easily observe that that max_accuracy = 100. **(b)** Zoom on the initial 5 epochs. The intercept clearly shows that the slowness value is $s = 1$.

Supplementary Figure 3: Catastrophic forgetting results with larger learning rate

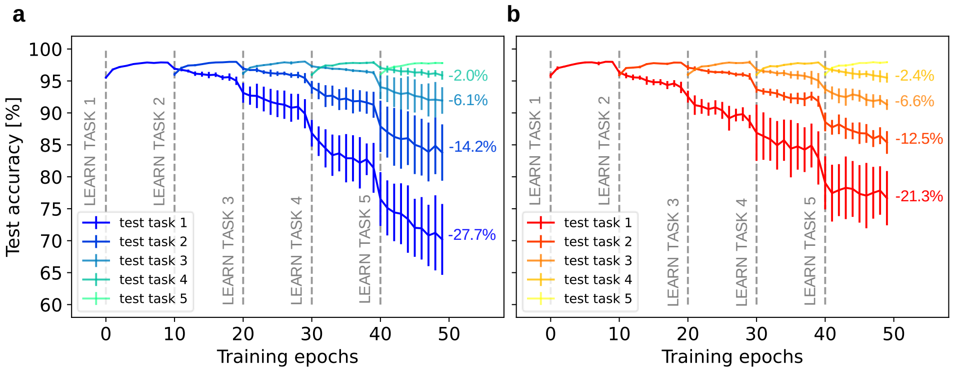

Supplementary Figure 3. Results for the permuted MNIST protocol with $\eta = 0.1$. **(a)** Test accuracy for SGD. **(b)** Test accuracy for GRAPES. The results are mean and standard deviation over 5 independent runs.

Supplementary Note 1: Choice of the range for the modulation factor

By design, the modulation factor for GRAPES is bounded in the interval [1,2]. This range has been empirically determined by varying the upper and lower bounds and choosing the interval that allows for the most significant improvements with respect to standard SGD. Supplementary Table **1** shows the test accuracy for different ranges of the modulation factor.

Range of modulation	Optimized learning rate	Test accuracy [%]
[1,2]	0.05	**98.53±0.06**
[1,3]	0.01	**98.53±0.06**
[1,5]	0.005	98.46±0.04
[0,2]	0.05	98.51±0.06
[0,5]	0.0005	98.43±0.05

Supplementary Table 1. **Test error on the MNIST dataset for networks trained with BP and SGD, with GRAPES modulation and different value range for the modulation factor**. The model architecture consists of 4 hidden layers, with 256 ReLU units each. The models are trained for 200 epochs with 10% dropout rate. The accuracy for each run is computed as the mean of the test accuracy over the last 10 training epochs. The reported result is mean and standard deviation over the accuracy of 10 independent runs. The best results are obtained with the modulation factor in the ranges $[1, 2]$ and $[1, 3]$.

Supplementary Note 2: Convergence analysis of GRAPES applied to SGD

We have shown that GRAPES implements a dynamic learning schedule for each weight. Here, we demonstrate the stability of GRAPES by analytically proving its convergence properties. The proof relies on the online learning framework proposed in [1], similarly to the investigation of the convergence of Adam optimizer in [2]. In online convex programming, an algorithm addresses a sequence of convex programming problems, each consisting of a convex feasible set $W \subseteq \mathbb{R}^n$, which is the same for all problems, and a convex cost function $f^t(w) : W \to \mathbb{R}$, which in principle is different for each problem. Given an arbitrary, unknown sequence of T such convex cost functions $f^1(w), f^2(w), ..., f^T(w)$, at each time step t the algorithm must predict the parameter vector w^t before observing the cost function f^t. After the vector w^t is selected, it is evaluated on f^t. Since the convex functions can be unrelated to one another and the nature of the sequence is unknown in advance, we evaluate the convergence of the GRAPES modulation using the regret function [1]. The regret function computes a difference between the proposed online algorithm and an "offline" algorithm. In the online algorithm, for each convex function a decision is made before the cost is known. On the other hand, the "offline" algorithm, prior to making a prediction, has knowledge about the sequence of convex cost functions, and makes a single choice to minimize the cost function $f(w) = \sum_{t=1}^{T} f^t(w)$. The regret function is defined as the difference between the cost of the online algorithm and the cost of the offline algorithm. It is computed as the sum of all the previous differences between the online prediction $f^t(w^t)$ and the best fixed point parameter $f^t(w^*)$:

$$R(T) = \sum_{t=1}^{T} [f^t(w^t) - f^t(w^*)] \tag{1}$$

where $w^* = \text{argmin}_{w \in W} \sum_{t=1}^{T} f^t(w)$.

We show that GRAPES modulation has $O(\sqrt{T})$ regret bound, similarly to standard SGD.

Theorem We express the learning rule obtained by applying the local GRAPES modulation to SGD as:

$$w^{t+1} = w^t - \eta^t M^t g^t \tag{2}$$

where:

- $g^t = \nabla f^t(w^t)$ is the gradient of the cost fuction with respect to the parameter w^t at time step t
- η^t is the learning rate at time step t
- M^t is the modulation factor at time step t. We define M_{max} as the maximum value obtained by the modulation factor. Due to the constraints of the modulation factor $M_{max} \leq 2$

Assume that the GRAPES modulation applied to SGD during training can select only the parameters w^i belonging to a convex feasible set W. Assume that the programming problem consists of an arbitrary, unknown sequence of convex cost functions $f^1(w), f^2(w), ..., f^T(w)$ such that $f^i(w) : W \to \mathbb{R}$. Assume that:

1. The feasible set is bounded,
   i.e., $\exists k \in \mathbb{R} : \forall w^i, w^j \in W$, $||w^i - w^j|| \leq k$
   We define $||W|| = \max_{w^i, w^j \in W} ||w^i - w^j||$
2. The feasible set is closed,
   i.e., $\forall \{w^1, w^2, ...\}$ where $w^t \in W$, $\forall t$,
   if $\exists w \in \mathbb{R}^n$ such that $w = \lim_{t \to \infty} w^t$, then $w \in W$
3. The feasible set is non-empty,
   i.e., $\exists w \in W$
4. The cost functions are differentiable, *i.e.*, $\forall t$, f^t is differentiable.
   We define the gradient of the cost function as $g^t = \nabla f^t(w^t)$
5. The cost functions have bounded gradients,
   i.e., $\exists k \in \mathbb{R} : \forall t, \forall w \in W, ||\nabla f^t(w)|| \leq k$
   We define $||\nabla f|| = \max_{w \in W, t \in \{1,2,...\}} ||\nabla f^t(w)||$

6. $\forall t, \forall w \in \mathbb{R}, \exists$ an algorithm A, so that, given w and $\nabla f^t(w)$, it can produce $\text{argmin}_{w \in W} ||w^i - w^j||$

Assume furthermore that the learning rate follows $\eta_t = t^{-1/2}$.

Under these assumptions the GRAPES modulation applied to SGD achieves the following guarantee, for all $T \geq 1$.

$$R(T) \leq \frac{||W||^2\sqrt{T}}{2M_T} + M_{max}||\nabla f||^2 \left(\sqrt{T} - \frac{1}{2}\right) \tag{3}$$

Corollary Under the same conditions of the Theorem, GRAPES modulation applied to SGD achieves the following guarantee, for all $T \geq 1$.

$$\frac{R(T)}{T} = O\left(\frac{1}{\sqrt{T}}\right) \tag{4}$$

Under the same conditions, standard SGD achieves the following guarantee, for all $T \geq 1$.

$$R_{SGD}(T) \leq \frac{||W||^2\sqrt{T}}{2} + ||\nabla f||^2 \left(\sqrt{T} - \frac{1}{2}\right) \tag{5}$$

and

$$\frac{R_{SGD}(T)}{T} = O\left(\frac{1}{\sqrt{T}}\right) \tag{6}$$

Main steps of the convergence analysis

First we show that, as f^t are convex functions, the regret function has an upperbound and can be expressed as

$$R(T) \leq \sum_{t=1}^{T}(w^t - w^*) \cdot g^t \tag{7}$$

Definition 1 For a convex feasible set W, a function $f : W \to \mathbb{R}$ is convex if for all $w^i, w^j \in W$, for all $\lambda \in [0, 1]$,

$$\lambda f(w^i) + (1 - \lambda)f(w^j) \geq f(\lambda w^i + (1 - \lambda)w^j) \tag{8}$$

Lemma 2 [1] If a function $f : W \to \mathbb{R}$ is convex, then for all $w^i, w^j \in \mathbb{R}$,

$$f(w^j) \geq f(w^i) + \nabla f(w^i)^\top(w^j - w^i) \tag{9}$$

Using Lemma 2 we have

$$f^t(w^t) - f^t(w^*) \leq (g^t)^\top(w^t - w^*) = \sum_{q=1}^{n} g_q^t(w_q^t - w_q^*), \tag{10}$$

where g_q^t, w_q^t and w_q^* are the k-th components of g^t, w^t and w^* respectively. This leads to

$$R(T) = \sum_{t=1}^{T}[f^t(w^t) - f^t(w^*)] \tag{11}$$

$$\leq \sum_{t=1}^{T} g^t \cdot (w^t - w^*) \tag{12}$$

Now we use the weight update rule $w^{t+1} = w^t - \eta^t M^t g^t$ and the learning rate dynamics $\eta_t = t^{-1/2}$ to show that

$$\frac{R(T)}{T} = O\left(\frac{1}{\sqrt{T}}\right) \tag{13}$$

We start by manipulating the weight update rule:

$$(w^{t+1}-w^*)^2 = (w^t - \eta^t M^t g^t - w^*)^2 = \tag{14}$$
$$= \left[(w^t - w^*) - \eta^t M^t g^t\right]^2 = \tag{15}$$
$$= (w^t - w^*)^2 - 2\eta^t M^t g^t (w^t - w^*) + (\eta^t)^2 (M^t)^2 (g^t)^2 \tag{16}$$

Since, by assumption, $||g^t|| = ||\nabla f^t|| \leq ||\nabla f||$, we can upper bound the expression above as:

$$(w^{t+1}-w^*)^2 \leq (w^t - w^*)^2 - 2\eta^t M^t g^t (w^t - w^*) + (\eta^t)^2 (M^t)^2 ||\nabla f||^2 \tag{17}$$

By rearranging we obtain:

$$\frac{\cancel{2\eta^t M^t} g^t (w^t - w^*)}{\cancel{2\eta^t M^t}} \leq \frac{(w^t - w^*)^2 - (w^{t+1}-w^*)^2 + (\eta^t)^2 (M^t)^2 ||\nabla f||^2}{2\eta^t M^t}$$
$$g^t (w^t - w^*) \leq \frac{1}{2\eta^t M^t}\left[(w^t - w^*)^2 - (w^{t+1}-w^*)^2\right] + \frac{\eta^t}{2} M^t ||\nabla f||^2 \tag{18}$$

By substituting equation 18 in equation 7 we can write:

$$\begin{aligned} R(T) &\leq \sum_{t=1}^{T} g^t \cdot (w^t - w^*) \\ &\leq \sum_{t=1}^{T} \left[\frac{(w^t - w^*)^2 - (w^{t+1}-w^*)^2}{2\eta^t M^t} + \frac{\eta^t}{2} M^t ||\nabla f||^2\right] \\ &\leq \sum_{t=1}^{T} \frac{(w^t - w^*)^2 - (w^{t+1}-w^*)^2}{2\eta^t M^t} + \frac{||\nabla f||^2}{2} \sum_{t=1}^{T} \frac{\eta^t}{2} M^t \\ &= \sum_{t=1}^{T} \frac{(w^t - w^*)^2}{2\eta^t M^t} - \sum_{t=1}^{T} \frac{(w^{t+1}-w^*)^2}{2\eta^t M^t} + \frac{||\nabla f||^2}{2} \sum_{t=1}^{T} \frac{\eta^t}{2} M^t \end{aligned} \tag{19}$$

We apply a change of variable to the second term of equation 19 as $t' = t+1$ to obtain:

$$\sum_{t=1}^{T} \frac{(w^{t+1}-w^*)^2}{2\eta^t M^t} = \sum_{t'=2}^{T+1} \frac{(w^{t'}-w^*)^2}{2\eta^{t'-1} M^{t'-1}} =$$
$$= \frac{(w^{T+1}-w^*)^2}{2\eta^T M^T} + \sum_{t'=2}^{T} \frac{(w^{t'}-w^*)^2}{2\eta^{t'-1} M^{t'-1}} \tag{20}$$

We then insert the result of equation 20 into equation 19:

$$R(T) \leq \left[\frac{(w^1 - w^*)^2}{2\eta^1 M^1} + \sum_{t=2}^{T} \frac{(w^t - w^*)^2}{2\eta^t M^t}\right] - \left[\frac{(w^{T+1}-w^*)^2}{2\eta^T M^T} + \sum_{t=2}^{T} \frac{(w^t - w^*)^2}{2\eta^{t-1} M^{t-1}}\right] + \tag{21}$$
$$+ \frac{||\nabla f||^2}{2} \sum_{t=1}^{T} \frac{\eta^t}{2} M^t = \tag{22}$$
$$= \frac{(w^1 - w^*)^2}{2\eta^1 M^1} - \frac{(w^{T+1}-w^*)^2}{2\eta^T M^T} + \frac{1}{2} \sum_{t=2}^{T} \left(\frac{1}{\eta^t M^t} - \frac{1}{\eta^{t-1} M^{t-1}}\right)(w^t - w^*)^2 + \tag{23}$$
$$+ \frac{||\nabla f||^2}{2} \sum_{t=1}^{T} \frac{\eta^t}{2} M^t \tag{24}$$

Now we use the assumption that the feasible set W is bounded, *i.e.*, $\forall t||w^t - w^*|| \leq ||W||$ to find:

$$R(T) \leq \frac{||W||^2}{2\eta^1 M^1} + \frac{1}{2}\sum_{t=2}^{T}\left(\frac{1}{\eta^t M^t} - \frac{1}{\eta^{t-1}M^{t-1}}\right)||W||^2 +$$
$$+ \frac{||\nabla f||^2}{2}\sum_{t=1}^{T}\eta^t M^t =$$
$$= \frac{||W||^2}{2}\left[\frac{1}{\eta^1 M^1} + \left(\frac{1}{\eta^2 M^2} - \frac{1}{\eta^1 M^1}\right) + ... + \left(\frac{1}{\eta^T M^T} - \frac{1}{\eta^{T-1}M^{T-1}}\right)\right] +$$
$$+ \frac{||\nabla f||^2}{2}\sum_{t=1}^{T}\eta^t M^t =$$
$$= \frac{||W||^2}{2}\frac{1}{\eta^T M^T} + \frac{||\nabla f||^2}{2}\sum_{t=1}^{T}\eta^t M^t \tag{25}$$

Next, we consider the assumptions $\eta_t = t^{-1/2}$ and $\forall t, M^t \in [1, 2)$.

$$\sum_{t=1}^{T}\eta^t M^t = \sum_{t=1}^{T}\frac{1}{\sqrt{t}}M^t$$
$$\leq 2\left(1 + \int_{t=1}^{T}\frac{dt}{\sqrt{t}}\right)$$
$$\leq 2\left(1 + \left[2\sqrt{t}\right]_1^T\right)$$
$$\leq 2\left(2\sqrt{T} - 1\right) \tag{26}$$

We then use equation 26 and the condition $\forall t, M^t \in [1, 2)$ in equation 25:

$$R(T) \leq \frac{||W||^2}{2}\frac{1}{\frac{M^T}{\sqrt{T}}} + \frac{||\nabla f||^2}{2}2\left(2\sqrt{T} - 1\right) \tag{27}$$
$$= \frac{||W||^2\sqrt{T}}{2M^T} + ||\nabla f||^2\left(2\sqrt{T} - 1\right) \tag{28}$$

Therefore,

$$\lim\sup_{t\to\infty}\frac{R(T)}{T} = \frac{\frac{||W||^2\sqrt{T}}{2M^T} + ||\nabla f||^2\left(2\sqrt{T} - 1\right)}{T} = 0 \tag{29}$$

Supplementary Note 3: Model performance with smaller learning rates than the optimized ones

Figures **4** and **5** show the performance of SGD and GRAPES on models trained with small learning rate $\eta = 0.001$. In Figure **4** we compare the performance of GRAPES and SGD on models of increasing complexity in terms of depth and layer size. In Figure **5** we show the performance of three training schemes (BP, FA, DFA) on different data sets (MNIST, Fashion MNIST, Extended MNIST, CIFAR-10).

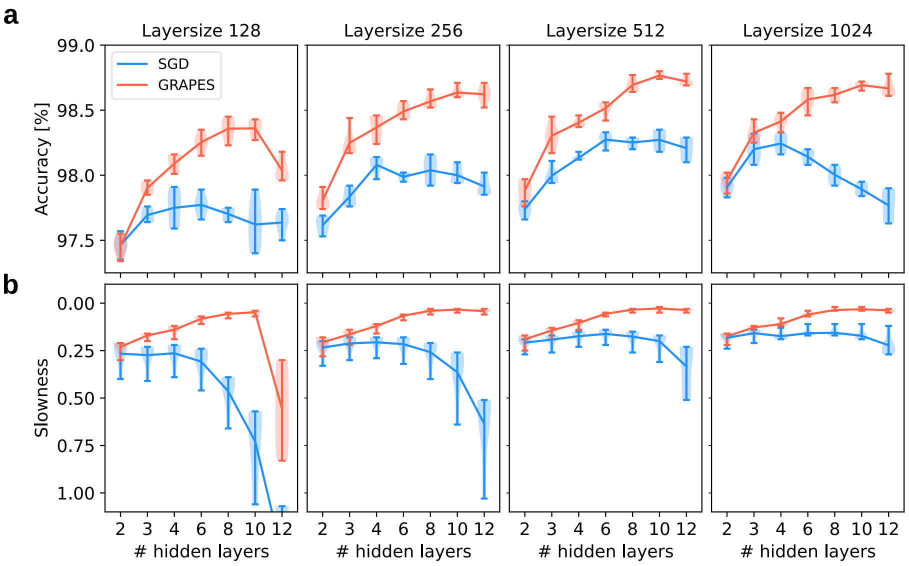

Supplementary Figure 4. (**a**) Test accuracy and (**b**) convergence rate in terms of slowness of networks with different layer size as a function of the number of hidden layers. The slowness parameter is computed by fitting the initial 100 epochs. The shaded area around the standard deviation bar shows the distribution of the results of five runs with a violin plot.

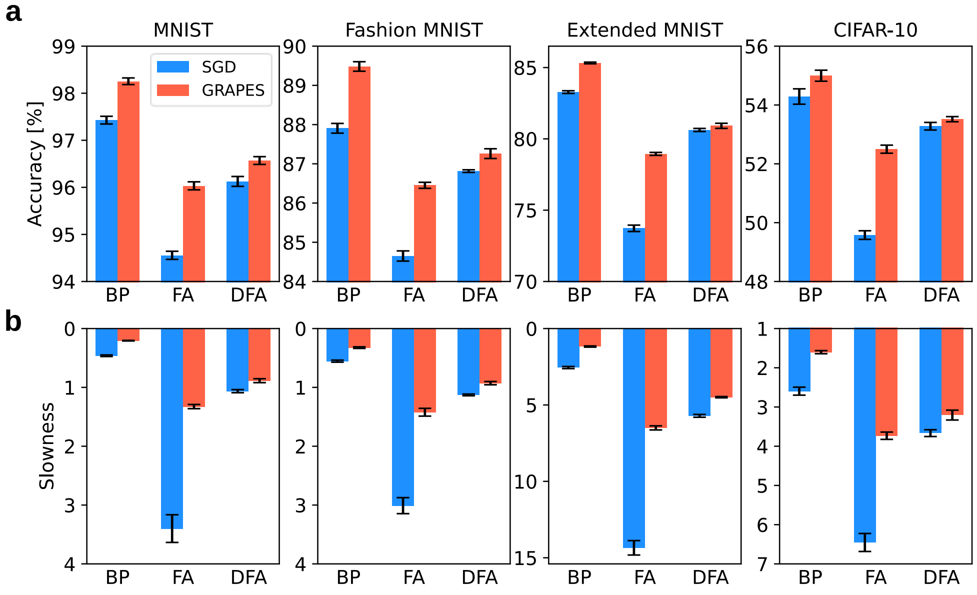

Supplementary Figure 5. (**a**) Test accuracy and (**b**) convergence rate in terms of slowness value for 3×256 ReLU networks, with 10% dropout, trained with BP, FA and DFA on MNIST, Fashion MNIST, Extended MNIST, and CIFAR-10 data sets. The slowness parameter is computed by fitting the initial 100 epochs. The results are mean and standard deviation over 5 independent runs.

Supplementary Note 4: Weight distribution after training with GRAPES

A further possible origin of the the benefits of GRAPES relies on the adjustment of the error signal. The inhomogeneous distribution of the local modulation factor, combined with the propagation to upstream layers in the propagating version, allows GRAPES to greatly enhance a subset of synaptic updates during training. Hence, small groups of synapses are enabled to strengthen or weaken their weights to a much larger extent with respect to SGD. We compared the layer-wise weight distribution of networks trained with SGD and with GRAPES, both initialized with a normal distribution. After training with SGD, the weight distribution is still close to a Gaussian. Instead, after GRAPES-optimization we observed that, particularly in the first hidden layers of deep networks, the weight distribution does not follow a Gaussian shape, is wider, and is characterized by long tails.

Recent electrophysiological studies have revealed that the amplitudes of EPSPs between cortical neurons are not distributed as Gaussians but obey a long-tailed pattern, typically lognormal [3, 4]. Such firing pattern implies that some synapses are very strong while many synapses are weak ("strong-sparse and weak-dense" networks) [5]. Heavy-tailed distributions were shown to lead to important network properties: faster transient responses, higher dynamical range and lesser sensitivity to random fluctuations in synaptic activity [6]. This suggests that the long-tail distribution found at many physiological and anatomical levels in the brain is fundamental to structural and functional brain organization [3]. From preliminary investigation, GRAPES seems to convey the network weights toward a more biologically plausible distribution. Fig. **6** illustrates this phenomenon. This implies that the properties of faster learning and greater robustness to noise exhibited by networks trained with GRAPES could stem from the modulation of the gradients towards adjusting the weights to a long-tailed distribution.

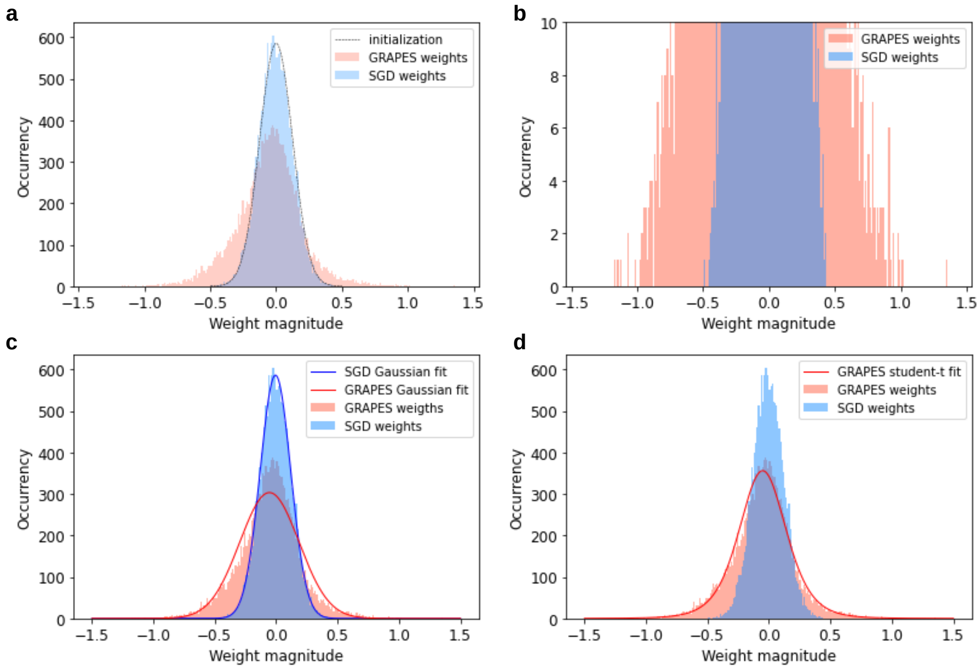

Supplementary Figure 6. Weight distribution and fit for the first hidden layer of a 12 hidden layer model. Hidden layer size is 128 and activation function is ReLU. (**a**) Distribution of the weights after training with SGD (red) or GRAPES (blue). The black dotted line shows the weight initialization. The weight distribution obtained with SGD remains significantly closer to the weight initialization than with GRAPES. (**b**) Zoom of panel (a) along the y axis. The weights trained with GRAPES show a longer tail and more outliers with respect to the weights trained with SGD. (**c**) The weights trained with SGD follow a normal distribution. The weights trained with GRAPES cannot be fitted well with a Gaussian shape. In particular the weights on the tail cannot be captured by the fit. (**d**) The weights trained with GRAPES have a distribution that follows more closely a student-t distribution than a Gaussian distribution. The student-t distribution belongs to the class of the heavy-tailed distributions, similarly to the lognormal distribution that has been experimentally measured in synaptic strength in neural circuits of mammalian brain.

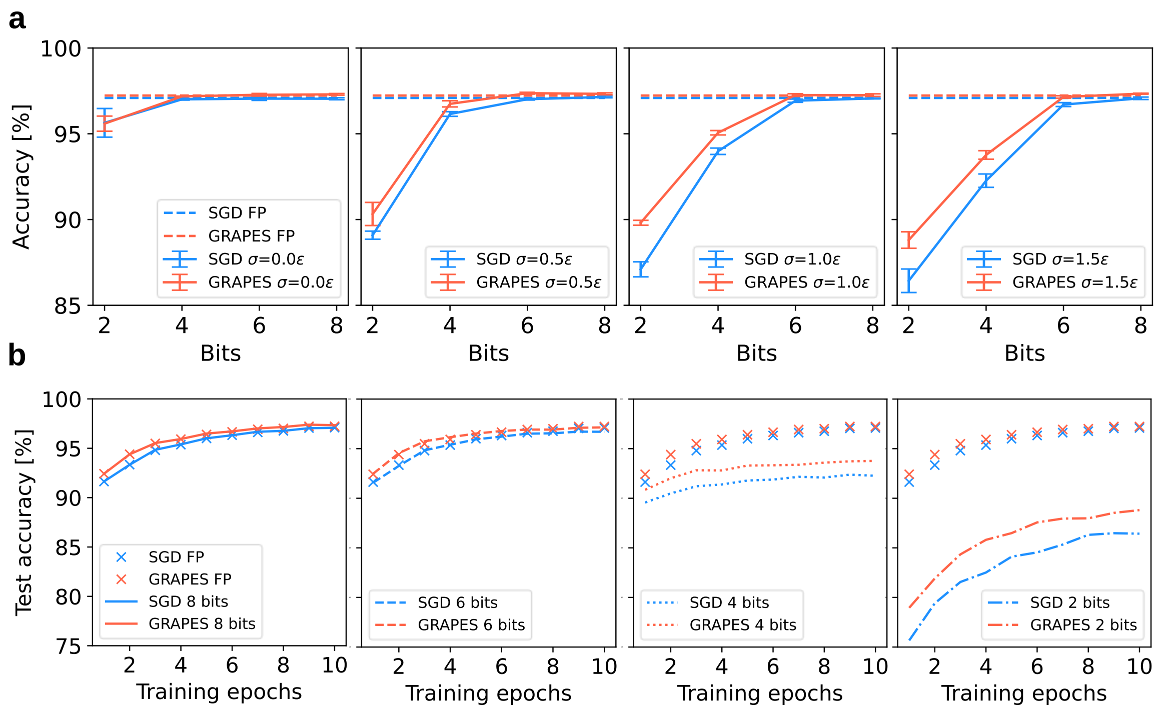

Supplementary Figure 7. (**a**) Final test accuracy for 1-hidden layer networks as a function of the n-bit granularity where $n \in \{2, 4, 6, 8\}$ and noise standard deviation $\sigma \in \{0.0, 0.5, 1.0, 1.5\}\epsilon$. The red curves correspond to networks trained with GRAPES, while the blue curves to networks trained with SGD. The results are mean and standard deviation over 5 independent runs. (**b**) Test curves for single run as a function of the training epochs with fixed noise standard deviation $\sigma = 1.5\epsilon$. Training with full precision (FP), 8,6,4 and 2 bits precision are compared.

Supplementary Note 5: Behaviour of GRAPES under hardware constraints

Training ANNs on hardware accelerators implies certain performance degradation due to a number of hardware-specific constraints, such as noisy synaptic updates, limited range of synaptic weights [7], and their update frequency and resolution [8]. We empirically demonstrate that GRAPES mitigates the accuracy degradation. Inspired by the approach in [9], we investigate the effect of granularity and stochasticity associated with weight-updates. First we establish a *reference* performance for a 1-hidden layer network, trained on the MNIST data set with full precision (FP) arithmetic (64-bits) and no noise. Specifically, after FP training, the network achieves a test accuracy of 97.08% with SGD and 97.23% with GRAPES. Then, we apply fixed n-bit granularity and stochasticity on the weight update as described below in *Simulation details*. Figure **7**(a) reports the classification accuracy for different granularity levels and noise amplitudes. In the absence of noise, the accuracy is close to the reference FP value for 4,6,8-bits precision, while for 2-bits granularity an accuracy drop of 1.5% is observed. As noise with increasing amplitude is added, the model accuracy progressively deteriorates. Such degradation is robustly mitigated when GRAPES is applied. Figure **7**(b) shows the test curve for a noise standard deviation of $\sigma = 1.5\epsilon$. For all weight granularities, GRAPES leads to a higher classification accuracy over the entire training period.

We remark that the implemented hardware constraints share many aspects with biological circuits: the synaptic transmission is affected by noise, the signal is quantized and neurons have a limited fan-in/fan-out. Interestingly, GRAPES retains many similarities with biological processes, such as synaptic integration, synaptic scaling and heterosynaptic plasticity. We therefore envision that the brain might exploit such mechanisms in order to overcome the limitations due to the mentioned constraints, and, in fact could turn out to endow the networks with the ability to take advantage of noise to improve the performance. Our analysis is consistent with previous work providing evidence that synaptic integration combined with the intrinsic noise stochasticity of Poisson trains enhances the computational capabilities of spiking networks on pattern classification tasks [10]. In conclusion, our findings suggest that incorporating GRAPES in on-chip training algorithms could pave the way for pivotal progress in learning algorithms for bio-inspired hardware and, in particular, for neuromorphic chips.

Simulation details We train 1-hidden layer networks on MNIST data set. The hidden layer has 250 sigmoid neurons, the output activation is softmax and the loss is cross-entropy. No dropout is introduced. The networks are trained for 10 training epochs with fixed learning rate $\eta = 0.4$. The weight distribution after training of the FP network lies in the range [-1,1]. To investigate the effect of fixed n-bit granularity, we assume a similar final weight distribution range as that from the floating-point simulation. Hence, we cover the weight range [-1,1] in $2^n - 2$ steps, with $n \in \{2, 4, 6, 8\}$. We use the n-bit granularity for the forward pass. We perform the backward pass on a floating point copy of the network and apply the granularity after the update. The stochasticity is applied on the weight update as white noise with mean zero and variance $\sigma = k\epsilon$, where $\epsilon = 1/(2^n - 2)$ and $k \in \{0.0, 0.5, 1.0, 1.5\}$. Both the final test accuracy and the test curves are averaged over five runs.

Supplementary Table 2: Comparison between local and propagating versions of GRAPES

Layers	Activation	DO	η	Train epochs	acc [%] SGD	acc [%] GRAPES prop	acc [%] GRAPES local	s SGD	s GRAPES prop	s GRAPES local
3 × 256	ReLU	0%	1e-3	200	97.46±0.09	**97.95±0.07**	97.80±0.04	0.36±0.02	**0.17±0.01**	0.27±0.02
3 × 256	ReLU	10%	1e-3	200	97.43±0.09	**98.25±0.08**	97.85±0.06	0.46±0.01	**0.21±0.01**	0.31±0.02
3 × 256	tanh	0%	1e-2	200	98.04±0.11	**98.20±0.07**	98.12±0.09	0.15±0.01	**0.08±0.00**	0.12±0.01
3 × 256	tanh	10%	1e-2	200	98.16±013	**98.42±0.07**	98.33±0.06	0.16±0.00	**0.10±0.00**	0.13±0.00

Supplementary Table 2. **Test accuracy and convergence rate on the MNIST dataset for networks trained with BP and SGD optimizer, comparing the results for the local and propagating version of GRAPES**. The reported result is the average and standard deviation of best test accuracy over five runs. "DO" stands for dropout, η is the learning rate. Both the local and the propagating versions of GRAPES always outperform the classic SGD both in terms of accuracy (acc) and slowness (s). The propagating version allows for the best improvements.

Supplementary Table 3: Results on MNIST data set with BP and SGD

Layers	Activation	DO	Def	Train epochs	acc [%] SGD	acc [%] GRAPES	acc [%] SGD augm η	s SGD	s GRAPES	s SGD augm η
3 × 256	ReLU	0%	No	200	97.46±0.09	**97.95±0.07**	97.81±0.06	0.36±0.02	**0.17±0.01**	0.27±0.01
3 × 256	ReLU	10%	No	200	97.43±0.09	**98.25±0.08**	97.90±0.09	0.46±0.01	**0.21±0.01**	0.32±0.02
3 × 256	ReLU	25%	No	200	97.15±0.09	**98.11±0.03**	97.81±0.09	0.55±0.04	**0.27±0.02**	0.38±0.04
10 × 256	ReLU	0%	No	600	97.21±0.12	**98.48±0.05**	97.34±0.08	0.22±0.02	**0.04±0.01**	0.14±0.01
10 × 256	ReLU	10%	No	600	98.16±0.06	**98.72±0.11**	98.18±0.07	0.99±0.05	**0.05±0.01**	0.59±0.06
10 × 256	ReLU	0%	Yes	600	98.81±0.06	**99.56±0.03**	99.01±0.07	1.27±0.09	**0.11±0.01**	0.78±0.05
10 × 512	ReLU	0%	No	300	97.41±0.06	**98.51±0.06**	97.50±0.12	0.16±0.01	**0.03±0.01**	0.11±0.01
10 × 512	ReLU	10%	No	300	98.11±0.12	**98.78±0.06**	98.19±0.04	0.60±0.06	**0.04±0.01**	0.33±0.02
10 × 512	ReLU	0%	Yes	300	98.43±0.18	**99.54±0.04**	98.82±0.05	0.84±0.08	**0.09±0.01**	0.53±0.04
3 × 256	tanh	0%	No	200	98.04±0.11	**98.20±0.07**	98.11±0.08	0.15±0.01	**0.08±0.00**	0.11±0.00
3 × 256	tanh	10%	No	200	98.16±013	**98.42±0.07**	**98.42±0.05**	0.16±0.00	**0.10±0.00**	0.13±0.00
3 × 256	tanh	25%	No	200	97.99±0.06	**98.37±0.03**	98.26±0.06	0.17±0.01	**0.12±0.00**	0.15±0.01
10 × 256	tanh	0%	No	600	98.10±0.07	**98.43±0.04**	98.15±0.10	0.09±0.001	**0.04±0.00**	0.07±0.00
10 × 256	tanh	10%	No	600	98.44±0.02	**98.62±0.01**	98.45±0.04	0.13±0.01	**0.06±0.00**	0.10±0.01
10 × 256	tanh	0%	Yes	600	99.33±0.04	**99.38±0.04**	99.35±0.06	0.32±0.02	**0.11±0.01**	0.22±0.01
10 × 512	tanh	0%	No	300	98.12±0.10	**98.56±0.05**	98.22±0.06	0.09±0.01	**0.04±0.00**	0.07±0.01
10 × 512	tanh	10%	No	300	98.33±0.04	**98.59±0.03**	98.40±0.06	0.11±0.01	**0.05±0.01**	0.09±0.01
10 × 512	tanh	0%	Yes	300	99.12±0.12	**99.37±0.08**	99.20±0.14	0.30±0.03	**0.10±0.01**	0.19±0.01

Supplementary Table 3. **Test accuracy and convergence rate on the MNIST dataset for networks trained with BP and SGD optimizer, with and without GRAPES modulation**. In the "Def" column we indicate whether the elastic deformation scheme [11] was used. "DO" stands for dropout. The learning rate is $\eta = 0.001$ with ReLU activation and $\eta = 0.01$ with tanh activation. The column marked with "augm η" indicate that the learning rate has been uniformly multiplied by a factor equal to the mean of the local modulation factor of GRAPES. The reported result is the average and standard deviation of the best test accuracy over five runs. The GRAPES modulation in most cases outperforms the classic SGD. Note that, even in the case where GRAPES is used with a smaller learning rate than SGD (network configuration with 10 hidden-layers and tanh activations, where $\eta = 0.01$ for SGD and $\eta = 0.001$ for GRAPES; see Supplementary Table 10 for details on the learning rate), it provides a better convergence rate.

Supplementary Table 4: Results on various data set with BP, FA, DFA

Data set	Train scheme	Layers	Activation	DO	Def	Train epochs	acc [%] SGD	acc [%] GRAPES	s SGD	s GRAPES
MNIST	FA	3 × 256	ReLU	10%	No	200	94.56±0.10	**96.03±0.10**	3.40±0.26	**1.33±0.04**
	FA	10 × 256	ReLU	10%	No	600	-	**91.30±0.48**	-	-
	FA	3 × 256	tanh	10%	No	200	97.09±0.03	**97.75±0.07**	0.22±0.01	**0.16±0.01**
	FA	10 × 256	tanh	10%	No	600	89.52±2.58	**94.54±0.74**	40.72±21.20	**1.63±0.94**
	DFA	3 × 256	ReLU	10%	No	200	96.13±0.12	**96.57±0.09**	1.06±0.03	**0.89±0.04**
	DFA	3 × 256	tanh	10%	No	200	**97.89±0.06**	97.87±0.04	0.17±0.01	**0.15±0.00**
	DFA	10 × 256	tanh	10%	No	300	97.93±0.05	**97.94±0.02**	0.19±0.01	**0.15±0.01**
Fashion MNIST	BP	3 × 256	ReLU	10%	No	200	87.91±0.14	**89.48±0.14**	0.55±0.02	**0.32±0.02**
	BP	10 × 256	ReLU	10%	No	600	89.21±0.23	**90.29±0.10**	1.06±0.10	**0.19±0.02**
	BP	3 × 256	tanh	10%	No	200	89.20±0.12	**89.97±0.13**	0.18±0.01	**0.15±0.01**
	BP	10 × 256	tanh	10%	No	600	89.84±0.08	**90.47±0.13**	0.16±0.01	**0.16±0.01**
	FA	3 × 256	ReLU	10%	No	200	84.65±0.15	**86.45±0.09**	3.01±0.15	**1.42±0.07**
	FA	10 × 256	ReLU	10%	No	600	-	**65.69±1.85**	-	**34.66±8.33**
	FA	3 × 256	tanh	10%	No	200	87.75±0.07	**88.70±0.08**	0.31±0.01	**0.20±0.01**
	FA	10 × 256	tanh	10%	No	600	**85.78±2.21**	85.76±1.98	3.03±1.62	**2.52±1.35**
	DFA	3 × 256	ReLU	10%	No	200	86.81±0.04	**87.26±0.14**	1.13±0.02	**0.93±0.03**
	DFA	3 × 256	tanh	10%	No	200	88.42±0.08	**88.53±0.08**	0.20±0.01	**0.17±0.01**
	DFA	10 × 256	tanh	10%	No	300	**88.89±0.04**	88.78±0.13	0.27±0.01	**0.19±0.01**
Extended MNIST	BP	3 × 256	ReLU	10%	No	200	83.28±0.10	**85.32±0.07**	2.55±0.08	**1.18±0.04**
	BP	10 × 256	ReLU	10%	No	400	84.08±0.21	**86.35±0.13**	5.46±0.30	**0.44±0.02**
	BP	3 × 256	tanh	10%	No	200	86.39±0.12	**86.40±0.12**	0.73±0.02	**0.38±0.01**
	BP	10 × 256	tanh	10%	No	400	**86.85±0.07**	86.39±0.12	**0.61±0.02**	1.44±0.09
	FA	3 × 256	ReLU	10%	No	200	73.73±0.25	**78.95±0.12**	14.35±0.52	**6.50±0.15**
	FA	10 × 256	ReLU	10%	No	400	-	**43.62±8.24**	-	**-**
	FA	3 × 256	tanh	10%	No	200	82.29±0.11	**83.74±0.09**	1.88±0.03	**1.07±0.02**
	FA	10 × 256	tanh	10%	No	300	71.58±0.86	**78.22±0.39**	22.73±0.75	**2.87±0.16**
	DFA	3 × 256	ReLU	10%	No	200	80.62±0.12	**80.91±0.20**	5.71±0.10	**4.49±0.05**
	DFA	3 × 256	tanh	10%	No	200	84.30±0.14	**84.36±0.15**	1.14±0.03	**0.87±0.02**
	DFA	10 × 256	tanh	10%	No	200	**83.95±0.11**	83.88±0.15	1.76±0.03	**1.05±0.02**
CIFAR-10	BP	3 × 256	ReLU	10%	No	600	54.29±0.29	**55.00±0.21**	2.60±0.11	**1.60±0.04**
	BP	10 × 256	ReLU	10%	No	500	42.18±0.44	**52.74±0.47**	13.09±5.81	**8.05±1.87**
	BP	3 × 256	tanh	10%	No	600	43.81±0.15	**49.43±0.17**	3.49±0.33	**1.82±0.13**
	BP	10 × 256	tanh	10%	No	500	42.77±0.34	**56.38±0.31**	4.12±0.59	**1.98±0.11**
	FA	3 × 256	ReLU	10%	No	400	49.58±0.16	**52.50±0.15**	6.45±0.26	**3.73±0.10**
	FA	3 × 256	tanh	10%	No	400	36.94±0.14	**42.45±0.34**	15.62±2.71	**10.35±0.39**
	FA	10 × 256	tanh	10%	No	400	-	**33.51±1.02**	-	**12.69±6.90**
	DFA	3 × 256	ReLU	10%	No	300	53.28±0.14	**53.52±0.10**	3.67±0.10	**3.21±0.14**
	DFA	3 × 256	tanh	10%	No	300	39.60±0.17	**41.47±0.12**	12.57±1.39	**10.93±1.54**
	DFA	10 × 256	tanh	10%	No	300	35.29±0.14	**39.58±0.20**	20.48±4.12	**16.64±1.14**

Supplementary Table 4. **Test accuracy and convergence rate of models with BP, FA and DFA learning schemes, obtained with and without GRAPES modulation on a variety of data sets**. The reported result is the average and standard deviation of the best test accuracy over 5 simulations. Empty fields in the accuracy columns indicate no convergence, while empty fields in the slowness columns indicate that the shape of the test curve could not be meaningfully fitted with the plateau equation. The learning rate is $\eta = 0.001$ with ReLU activation and $\eta = 0.01$ with tanh activation. The number of training epochs for each simulation is set based on the saturation of the models' performance. The GRAPES modulation in most cases outperforms the classic SGD.

Supplementary Table 5: Results on MNIST data set with BP and RMSprop

Model	Activation	Results in [12]	RMSprop	GRAPES + RMSprop
7 × 240	tanh	2.16±0.13%	**2.05±0.09** %	**2.05±0.10%**
1 × 800	tanh	1.59±0.04%	1.53±0.08%	**1.50±0.04%**
2 × 800	tanh	1.60±0.06%	1.51±0.05 %	**1.47±0.05 %**
3 × 800	tanh	1.75±0.05%	1.65±0.07 %	**1.62±0.04%**
4 × 800	tanh	1.92±0.11%	1.86±0.06 %	**1.79±0.06%**
2 × 800	sigmoid	1.67±0.03 %	**1.56±0.06 %**	1.62±0.05 %
2 × 800	ReLU	1.48±0.06%	**1.50±0.05%**	**1.50±0.06%**

Supplementary Table 5. **Test error on the MNIST dataset for networks trained with BP and RMSprop optimizer on the settings from [12], with and without GRAPES modulation**. The reported result is the mean and standard deviation over the final test accuracy of 10 runs. The GRAPES modulation in most cases outperforms the classic RMSprop.

Supplementary Table 6: Results on MNIST data set with FA and RMSprop

Model	Activation	Results in [12]	RMSprop	GRAPES + RMSprop
7 × 240	tanh	2.20±0.13%	3.34±0.10%	**2.56±0.13**%
1 × 800	tanh	1.68±0.05%	1.65±0.06%	**1.61±0.04**%
2 × 800	tanh	1.64±0.03%	1.67±0.01%	**1.65±0.04**%
3 × 800	tanh	1.66±0.09%	**1.57±0.07** %	1.60±0.07 %
4 × 800	tanh	1.70±0.04%	1.79±0.07%	**1.64±0.08**%
2 × 800	sigmod	1.82±0.10%	**1.78±0.07** %	1.80±0.03%
2 × 800	ReLU	1.74±0.10%	**1.66±0.02**%	1.69±0.05%

Supplementary Table 6. **Test error on the MNIST dataset for networks trained with FA and RMSprop optimizer on the settings from [12], with and without GRAPES modulation**. The reported result is the mean and standard deviation over the final test accuracy of 5 runs. The GRAPES modulation in most cases outperforms the classic RMSprop.

Supplementary Table 7: Results on MNIST data set with DFA and RMSprop

Model	Activation	Results in [12]	RMSprop	GRAPES + RMSprop
7 × 240	tanh	2.32±0.15%	2.20±0.07%	**2.11±0.05**%
2 × 800	tanh	1.74±0.08%	1.65±0.04%	**1.64±0.03**%
3 × 800	tanh	1.70±0.04%	**1.64±0.08**%	1.65±0.07%
4 × 800	tanh	1.83±0.07%	**1.69±0.04**%	**1.73±0.07**%
2 × 800	sigmod	1.75±0.04%	**1.84±0.06**%	1.85±0.04%
2 × 800	ReLU	1.70±0.06%	1.71±0.07%	**1.64±0.06**%

Supplementary Table 7. **Test error on the MNIST dataset for networks trained with DFA and RMSprop optimizer on the settings from [12], with and without GRAPES modulation**. The reported result is the mean and standard deviation over the final test accuracy of 5 runs. The GRAPES modulation in most cases outperforms the classic RMSprop.

Supplementary Table 8: Results on MNIST data set with BP and NAG

Model	Activation	Dropout	Results in [13]	NAG	GRAPES + NAG
1 × 500	tanh	0.0	1.72±0.08%	1.71±0.003%	**1.68±0.003**%
1 × 500	tanh	0.1	1.55±0.03%	1.69±0.02%	**1.64±0.02**%
1 × 500	tanh	0.25	1.64±0.06%	1.70±0.02%	**1.63±0.02**%
1 × 1000	tanh	0.0	1.76±0.06%	**1.69±0.003**%	1.72±0.002%
1 × 1000	tanh	0.1	1.58±0.03%	1.68±0.02%	**1.63±0.02**%
1 × 1000	tanh	0.25	1.70±0.06%	1.68±0.03%	**1.62±0.03**%
2 × 500	tanh	0.0	1.62±0.12%	**1.56±0.003**%	1.61±0.004%
2 × 500	tanh	0.1	1.61±0.05%	1.62±0.02%	**1.57±0.02**%
2 × 500	tanh	0.25	1.84±0.05%	1.76±0.02%	**1.60±0.02**%
2 × 1000	tanh	0.0	1.67±0.07%	1.58±0.002%	**1.50±0.002**%
2 × 1000	tanh	0.1	1.85±0.06%	1.77±0.04%	**1.49±0.01**%
2 × 1000	tanh	0.25	2.31±0.06%	2.04±0.04 %	**1.62±0.02**%

Supplementary Table 8. **Test error on the MNIST dataset for networks trained with BP and NAG optimizer on the settings from [13], with and without GRAPES modulation**. The accuracy for each run is computed as the mean of the test accuracy over the last 10 training epochs. The reported result is the mean and standard deviation over the accuracy of 10 independent runs. The GRAPES modulation in most cases outperforms the classic NAG.

Supplementary Table 9: Results on CIFAR-10 and CIFAR-100 datasets with residual networks, BP and Adam

	CIFAR-10		CIFAR-100	
Optimizer	Accuracy [%] $\eta = 1e-3$	Accuracy [%] $\eta = 1e-1$	Accuracy [%] $\eta = 1e-3$	Accuracy [%] $\eta = 1e-1$
Adam	76.07	83.08	45.52	54.98
Adam+ GRAPES	76.96	80.25	44.65	52.65

Supplementary Table 9. **Test accuracy on the CIFAR-10 and CIFAR-100 datasets for residual networks trained with BP and Adam optimizer, with and without GRAPES modulation, for learning rates larger and smaller than the optimized one** . We trained the models with learning rates $\eta = 1e-3$ and $\eta = 1e-1$, respectively smaller and larger than the optimized learning rate $\eta = 1e-2$. The results confirm that $\eta = 1e-2$ is the optimal learning rate for both SGD and GRAPES. The reported results are for a single run.

Supplementary Table 10: Models and hyperparameter settings for all experiments

Experiment	Figure/ Table	Training scheme	Dataset	Model	Opt.	Act.	DO [%]	bs	#ep.	#trials	η SGD	η GRAPES
M^l Dynamics	Fig.3	BP	MNIST	input layer: 784 × 256<br>10 hidden fc: 256 × 256<br>output layer: 256× 10	SGD	ReLU	0.1	64	200	1	0.001	0.001
Slowness fit	Fig.4a	BP	MNIST	input layer: 784 × 256<br>10 hidden fc: 256 × 256<br>output layer: 256× 10	SGD	ReLU	0.1	64	200	1	0.001	0.001
Scalability	Fig.4b,c	BP	MNIST	input layer: 784 × *ls*<br>3 hidden fc: *ls* × *ls*<br>output layer: *ls*× 10<br>$ls \in \{256, 400, 512, 800\}$	SGD	ReLU	0.1	64	200	10	$\in [0.001, 0.5]$	$\in [0.001, 0.1]$
Scalability	Fig.5a,b	FA	Extended MNIST	input layer: 784 × *ls*<br>3 hidden fc: *ls* × *ls*<br>output layer: *ls*× 10<br>$ls \in \{256, 400, 512, 800\}$	SGD	ReLU	0.1	64	200	10	$\in [0.001, 0.5]$	$\in [0.001, 0.5]$
Scalability	Fig.5c,d	DFA	Fashion MNIST	input layer: 784 × *ls*<br>3 hidden fc: *ls* × *ls*<br>output layer: *ls*× 10<br>$ls \in \{256, 400, 512, 800, 1024\}$	SGD	ReLU	0.1	64	200	10	$\in [0.001, 0.1]$	$\in [0.001, 0.1]$
Convolutional residual model	Tab.1	BP	CIFAR-10, CIFAR-100	As in [14], except without the max pool after conv5 for better accuracy	Adam	ReLU	None	256	250	10	0.01	0.01
Catastrophic forgetting (Avalanche)	Fig.6a,b	BP	Permuted MNIST	input layer: 784 × 256<br>1 hidden fc: 256 × 256<br>output layer: 256× 10	mom	ReLU	None	64	1	10	0.001	0.001
Catastrophic forgetting (numpy)	Fig.6c-f	BP	Permuted MNIST	input layer: 784 × 256<br>2 hidden fc: 256 × 256<br>output layer: 256× 10	SGD	ReLU	0.1	64	10	5	0.001	0.001
Catastrophic forgetting (numpy)	Fig.S3	BP	Permuted MNIST	input layer: 784 × 256<br>2 hidden fc: 256 × 256<br>output layer: 256× 10	SGD	ReLU	0.1	64	10	5	0.1	0.1
SNU	Fig.7a	BPTT	Rate MNIST	input layer: 784 × *ls*<br>2 hidden fc: *ls* × *ls*<br>output layer: *ls*× 10<br>$ls \in \{256, 512\}$	SGD	Step	None	64	200	5	0.1	0.1
sSNU	Fig.7a	BPTT	Rate MNIST	input layer: 784 × *ls*<br>1 hidden fc: *ls* × *ls*<br>output layer: *ls*× 10<br>$ls \in \{256, 512\}$	SGD	sigmoid	None	64	200	5	0.2	0.2
Hardware constraints	Fig.S5	BP	MNIST	input layer: 784 × 250<br>1 hidden fc:<br>output layer: 250× 10<br>$ls \in \{256, 512\}$	SGD	sigmoid	None	64	10	5	0.4	0.4

Supplementary Table 10. **Model and hyperparameter settings for all experiments**.

SUPPLEMENTARY REFERENCES

[1] Zinkevich, M. Online convex programming and generalized infinitesimal gradient ascent. In *Proceedings of the Twentieth International Conference on International Conference on Machine Learning*, ICML'03, 928–935 (AAAI Press, 2003).
[2] Kingma, D. P. & Ba, J. Adam: A method for stochastic optimization. In *ICLR (Poster)* (2015).
[3] Buzsáki, G. & Mizuseki, K. The log-dynamic brain: How skewed distributions affect network operations. *Nature reviews. Neuroscience* **15**, 264–278 (2014).
[4] Song, S., Sjöström, P. J., Reigl, M., Nelson, S. & Chklovskii, D. B. Highly nonrandom features of synaptic connectivity in local cortical circuits. *PLOS Biology* **3**, e68 (2005).
[5] Teramae, J. & Fukai, T. Computational implications of lognormally distributed synaptic weights. *Proceedings of the IEEE* **102**, 500–512 (2014).
[6] Iyer, R., Menon, V., Buice, M., Koch, C. & Mihalas, S. The influence of synaptic weight distribution on neuronal population dynamics. *PLOS Computational Biology* **9**, 1–16 (2013).
[7] Youhui Zhang, Yu Ji, Wenguang Chen & Yuan Xie. Neural network transformation under hardware constraints. In *2016 International Conference on Compliers, Architectures, and Sythesis of Embedded Systems (CASES)*, 1–1 (2016).
[8] Pfeil, T. *et al.* Is a 4-bit synaptic weight resolution enough? – constraints on enabling spike-timing dependent plasticity in neuromorphic hardware. *Frontiers in Neuroscience* **6**, 90 (2012). URL `https://www.frontiersin.org/article/10.3389/fnins.2012.00090`.
[9] Nandakumar, S. R. *et al.* Mixed-precision architecture based on computational memory for training deep neural networks. In *2018 IEEE International Symposium on Circuits and Systems (ISCAS)*, 1–5 (2018).
[10] Li, X., Luo, S. & Xue, F. Effects of synaptic integration on the dynamics and computational performance of spiking neural network. *Cognitive Neurodynamics* **14**, 347–357 (2020).
[11] Cireşan, D. C., Meier, U., Gambardella, L. M. & Schmidhuber, J. Deep, big, simple neural nets for handwritten digit recognition. *Neural Computation* **22**, 3207–3220 (2010).
[12] Nokland, A. Direct feedback alignment provides learning in deep neural networks. In *Proceedings of the 30th International Conference on Neural Information Processing Systems*, NIPS'16, 1045–1053 (Curran Associates Inc., Red Hook, NY, USA, 2016).
[13] Frenkel, C., Lefebvre, M. & Bol, D. Learning without feedback: Direct random target projection as a feedback-alignment algorithm with layerwise feedforward training (2019). Preprint at https://arxiv.org/abs/1909.01311.
[14] M., W. cifar10-resnet. `https://github.com/matthias-wright/cifar10-resnet` (2019).
